# Case of Cerebral Abscess Following Slight Injury

**Published:** 1884-06

**Authors:** A. N. Godby Gibbs

**Affiliations:** House Surgeon, Children's Hospital, Bristol


### CASE OF CEREBRAL ABSCESS FOLLOWING SLIGHT INJURY.

By A. N. Godby Gibbs, House Surgeon, Children's Hospital, Bristol.

George Hooper, æt. 7, was admitted into the Children's Hospital on Thursday, Jan. 3rd, 1884.

*History.*—Until three weeks before admission was a very healthy child. On Dec. 13th, three weeks ago, whilst at play in the street, he ran violently against a piece of iron on a trolly, causing a small contused wound on the upper part of the forehead, a little to the left of the middle line. He was not stunned, nor did he vomit, but ran home, and then with his father walked up to the Infirmary, where the wound was dressed; and as he showed no signs of serious mischief to the skull or brain, was sent home to attend as an out-patient. Except for slight headache, he seemed to do very well for a fortnight after the accident; was quite lively, seemed in his usual health, and even went to school. The wound nearly healed. On Saturday, Dec. 29th (sixteen days after the accident and five before admission into the Hospital), he began to vomit, and continued to do so at frequent intervals till he was admitted: at this time also he complained of severe headache. He had no convulsions or paralysis, had not squinted, and was quite conscious, except for slight wandering at night.

*Condition on admission* (twenty-one days after accident).—The body was somewhat emaciated. The boy had a pinched look, and his eyes were heavy. He was conscious, but rather apathetic, answering slowly. There was no paralysis nor squinting. He was vomiting bilious fluid at intervals, and complained of severe headache. The pupils were rather contracted, equal, and sluggish. The tongue was very furred. Temperature 99°; pulse 64. The wound on the forehead was scabbed over and almost healed; there was no inflammation of the surrounding skin.

*Progress.*—Jan. 6th (three days after admission). The vomiting had become less frequent; he was conscious, but dozed a good deal, screaming out occasionally in his sleep, and was very impatient if the nurse did not go to him the moment he called. The wound was suppurating a little.

Jan. 10th (one week after admission). He had improved a great deal. There was scarcely any headache. The vomiting had ceased. The boy was quite bright and lively, wanting to sit up.

Jan. 12th (the day before his death). Vomiting reappeared, as also the drowsiness, though he was quite conscious, and free from paralysis and convulsions. Temperature, 98·6°.

Jan. 13th. After a quiet night, seemed no worse early in the morning, when he talked to the nurse quite sensibly; he then had full power in his limbs. At 9 a.m. he began to vomit, with a good deal of straining; after this he got very faint, but revived after some brandy. At 10 a.m. he had the first convulsion since the accident; others followed rapidly, he became comatose, and died at 3 p.m. The convulsions were general, and affected both sides equally. There was no squinting.

*Post-mortem Examination.*—There was a partly-healed wound, about $\frac{3}{4} \times \frac{1}{2}$ inch, near centre of forehead. There was no effusion of blood round. On removing the scalp the bone at the seat of injury was found stripped of periosteum and rather porous. There was no fracture. The skull-cap, which was rather thin, having been removed, some lymph was seen effused on the surface of the dura mater, at a point corresponding with the external wound, and the inner surface of the bone was also inflamed. The whole thickness of the bone would probably have become necrosed. After the dura mater had been removed, the surface of both hemispheres looked healthy. In the anterior and upper part of the left hemisphere there was a large abscess containing yellow pus without admixture of blood. The abscess cavity was as large as a Tangerine orange. The wall was slightly thickened, and at the upper part only about a quarter of an inch of brain substance remained. The rest of the brain and other organs were healthy. No fracture of the base of the skull was found.

*Remarks.*—1. The boy for sixteen days after the injury went about as usual, and showed no sign of serious brain mischief; he even went to school.

2. After the first onset of symptoms he improved so much as to seem in a fair way to recovery, and only became very bad a few hours before death.

3. The temperature was never above 99°.

4. His mental condition was perfectly clear, his speech perfect twelve hours before death, and he had up to then had no paralysis or convulsion, though at that time fully one-fourth of the left hemisphere of the cerebrum must have been transformed into an abscess.

This case, I think, is interesting as showing that severe destructive brain mischief, after an apparently slight injury, may be proceeding for a considerable time without giving rise to symptoms; also that such mischief may be at some distance from the surface of the brain, and be covered by healthy brain tissue. This last point is especially noteworthy with reference to possible operative interference.

